# A Description of Mortality Associated with IPT plus ART Compared to ART Alone among HIV-Infected Individuals in Addis Ababa, Ethiopia: A Cohort Study

**DOI:** 10.1371/journal.pone.0137492

**Published:** 2015-09-08

**Authors:** Dumessa Edessa, Jimma Likisa

**Affiliations:** 1 Haramaya University, College of Health and Medical Sciences, Department of Pharmacology and Clinical Pharmacy, Harar, Ethiopia; 2 Ambo University, College of Medicine and Health Sciences, Department of Pharmacy, Clinical Pharmacy Unit, Ambo, Ethiopia; University of Pennsylvania School of Medicine, UNITED STATES

## Abstract

**Background:**

Tuberculosis (TB) is the most common human immunodeficiency virus (HIV) associated opportunistic infection. It is the leading cause of death in HIV-infected individuals in sub-Saharan Africa. Anti-retroviral therapy (ART) and isoniazid preventive therapy (IPT) are the two useful TB preventative strategies available to reduce TB among people living with HIV (PLHIV). Therefore, the aim of this study is to compare mortality associated with IPT taken together with ART, as well as ART alone, among PLHIV.

**Methods:**

A retrospective cohort study was undertaken at Tikur Anbessa Specialized Hospital (TASH) and Zewditu Memorial Hospital (ZMH) on 185 patients receiving IPT (6 months) plus ART and 557 patients receiving ART alone. Mortality rates (MR) per 100 person-years (PYs) were used to compare mortality rates amongst the groups. Time-to-death and survival probabilities of the patients were determined using the Kaplan Meier Method. The Cox Proportional Hazard Model was employed to estimate the effect of IPT plus ART on survival of PLHIV.

**Results:**

The mortality cases noted in patients treated by IPT plus ART versus ART alone were 18 (4.5 cases/100 PYs) and 116 (10 cases/100 PYs), respectively. In reference to the ART alone, the IPT plus ART reduced the likelihood of death significantly (aHR 0.48; 95% CI 0.38–0.69) and median time to death was about 26 months (IQR 19–34). Moreover, WHO stage IV (aHR 2.42: 95% CI 1.42–4.11), CD4 values ≥350cells/mm3 (aHR 0.52; 95% CI 0.28–0.94), adherence to ART (aHR 0.12; 95% CI 0.08–0.20), primary levels of education (aHR 2.20; 95% CI 1.07–4.52); and alcohol consumption (aHR 1.71; 95% CI 1.04–2.81) were factors strongly associated with mortality.

**Conclusion:**

We found that PLHIV treated by the IPT plus ART had a lower likelihood of mortality and delayed time-to-death when compared to patients treated by ART alone.

## Introduction

Tuberculosis (TB) is the most common human immunodeficiency virus (HIV) related opportunistic diseases and is the leading cause of death in HIV infected individuals in sub-Saharan Africa [1, 2]. According to the World Health Organization (WHO) estimation, one-third of the world population is latently infected with *mycobacterium tuberculosis* (MTB) which could reactivate later on. The risk of reactivation of the latent infection to active disease is greatly increased in people living with HIV (PLHIV) [3, 4]. In addition, HIV infection could also increase the risk of rapid progression of primary TB [5]. Thus, HIV infection and TB are synergistic infectious diseases [6] implying a need for well planned prevention or treatment of one of the infections in order to reduce the burden of the other infection.

The risk of developing TB is about 20–37 times greater in PLHIV than in those who are not HIV infected, making HIV infection the strongest risk factor for developing tuberculosis in those with latent or a new MTB infection [4]. The progression of latent TB infection to active disease during the lifetime of the general population is about 10%, whereas PLHIV who are infected with MTB the lifetime risk is about 30% [6–8]. This implies that TB and HIV accelerates the decline of immunological functions leading to subsequent death if left untreated [6].

Therefore, prevention of latent TB infection reactivation, and new TB infection, is one of the most important measures that may help in reducing the morbidity and mortality associated with HIV infection [9]. Even with the advent of Anti-retroviral Therapy (ART), PLHIV who develop TB have a manifold increased risk of death, which may reach as high as 15–20% [1]. Recent studies have also shown that isoniazid preventive therapy (IPT) could be an added intervention to reduce latent TB progression to active TB in PLHIV. However, all-cause mortality reduction in this patient group is not evidenced [7, 9]. The use of IPT, together with ART, in PLHIV is associated with a significant reduction in tuberculosis [9]. Although the TB-related mortality benefits were revealed by randomized controlled trials of IPT, all-cause mortality reduction was not significantly shown [10]. Nevertheless, most of the randomized controlled trials of IPT included in recent meta-analysis did not involve ART and hence all-cause mortality benefits were not shown [11].

Globally, more than 75% of estimated HIV associated incident tuberculosis cases live in just 10 countries, which include Ethiopia [2]. Throughout 2004–2012, it has been estimated that tuberculosis related deaths among people living with HIV was reduced by 25–50% in Ethiopia [2]. Nonetheless, this effort at mortality reduction is not sufficient for tuberculosis, which is curable and entirely preventable even in PLHIV. ART and IPT are the two useful TB preventive strategies available and their wider use is recommended by the WHO, American Thoracic Society (ATS) and Centers for Disease Control and Prevention (CDC) [12]. However, coverage and uptake of these preventive strategies are not 100% [2]. Beside this constraint, data that describes the association of IPT plus ART, and ART alone, on the rate of mortality among PLHIV on chronic HIV care follow-up are very limited. In addition, the applicability of the data obtained from other countries in the Ethiopian context could have limitations due to the variability of clinical cohorts in different nations. Hence, the primary aim of the present study is to identify whether or not IPT plus ART, in fact, lowers the rate of all-cause mortality more than the independent effect of ART among PLHIV.

## Methods

### Study Site and Design

This study was undertaken at ART clinics in two hospitals in Addis Ababa, namely Tikur Anbessa Specialized Hospital (TASH) and Zewditu Memorial Hospital (ZMH). The sites have existing program for the prevention of tuberculosis as well as HIV treatment. The study was a cohort study design primarily comparing mortality effects associated with IPT plus ART versus ART alone in PLHIV on chronic HIV care follow-up. The timing of IPT plus ART was six (6) months and the patients were followed-up while remaining on the ART until occurrence of the outcome or date of administrative censoring.

### Eligibility to ART and/or IPT

In Ethiopia, provided that there is no active TB and other co-morbidities, all adolescents and adults including pregnant women with HIV infection and CD4 counts ≤ 350 cells/mm^3^ are eligible to start ART regardless of the presence or absence of clinical symptoms. Those with severe or advanced clinical disease (WHO clinical stage III or IV) are eligible to start ART irrespective of their CD4 cell count.

The regimens commonly used are classified as preferred first-line regimens (TDF/3TC/EFV, ZDV/3TC/EFV and ZDV/3TC/NVP), alternative first-line regimens (d4T/3TC/EFV, d4T/3TC/EFV, TDF/3TC/NVP, d4T/3TC/NVP, ABC/3TC/EFV, ABC/3TC/NVP and ABC/3TC/ZDV) and second-line regimens (ZDV ± 3TC+LPV/r or ATV/r, ZDV/ABC+LPV/r or ATV/r, TDF/3TC ± ZDV+LPV/r or ATV/r, ABC/ddI +LPV/r or ATV/r and EFV or NVP/LPV/r or ATV/r). Treatment outcome monitoring practices in the country are six monthly CD4 cell count, viral load determination (as necessary), three monthly hemoglobin level determinations and other laboratory value evaluations that include liver and renal function tests [13].

Eligibility criteria for IPT in HIV infection, however, are not targeted because tuberculin skin testing (TST) is not in practice to identify the presence or absence of latent TB infection. Clinical practice in the country with regard to initiating IPT depends on results of symptom screening, WHO Clinical stage of the patient, and whether there is contraindication for IPT or not. PLHIV who are completely without symptoms compatible with TB (cough, fever, weight loss, etc.) can be given IPT after the absence of symptoms is confirmed. Those with symptoms should be evaluated with appropriate tests, such as sputum smear and culture. And those who are found to have TB should be offered TB therapy while those cases where TB is ruled out IPT should be offered. Any one-or-more conditions, like active tuberculosis, symptoms compatible with tuberculosis (even if the diagnosis of TB cannot be confirmed), abnormal chest X-ray; diagnosis and treatment of TB in the past 3 years; poor prognosis (terminally ill AIDS patients), history of poor compliance with treatment, active hepatitis (chronic or acute), known or reported high daily alcohol consumption, prior allergy or intolerance to isoniazid and history of close contact with multiple drug resistance TB (MDR-TB) patients are contraindications to receive IPT [14]. For these reasons, all non-contraindicated HIV positive individuals are eligible to receive IPT but only some individuals were provided with IPT (in addition to ART). However, active TB should be excluded first based on the clinical or laboratory investigations prior to initiation of IPT.

### Inclusion and exclusion criteria

HIV-infected adult patients (age ≥ 18 years old) eligible for inclusion in this analysis were those who had made two or more visits to the hospital clinic between September 11, 2009 and March 31, 2012, and those patients who received their primary care (ART or IPT plus ART) from the clinics. Patients who were on anti-tuberculosis treatment before admission into the study, or those patients who had elevated/altered liver function tests (LFTs) (value 5 times above the normal limits in asymptomatic patients or 3 times above the normal limits in symptomatic patients) at baseline, and those patients neither on IPT and ART nor on ART alone were excluded. Additionally, PLHIV who began with ART, but did not successfully complete IPT (adherence rate of less than 85% or ≥ 6 missed doses out of 30 doses), were excluded from the IPT plus ART group. Patients for whom the date (month and year) of starting treatment was unknown were also excluded.

### Selection of study participants

**Fig 1:**
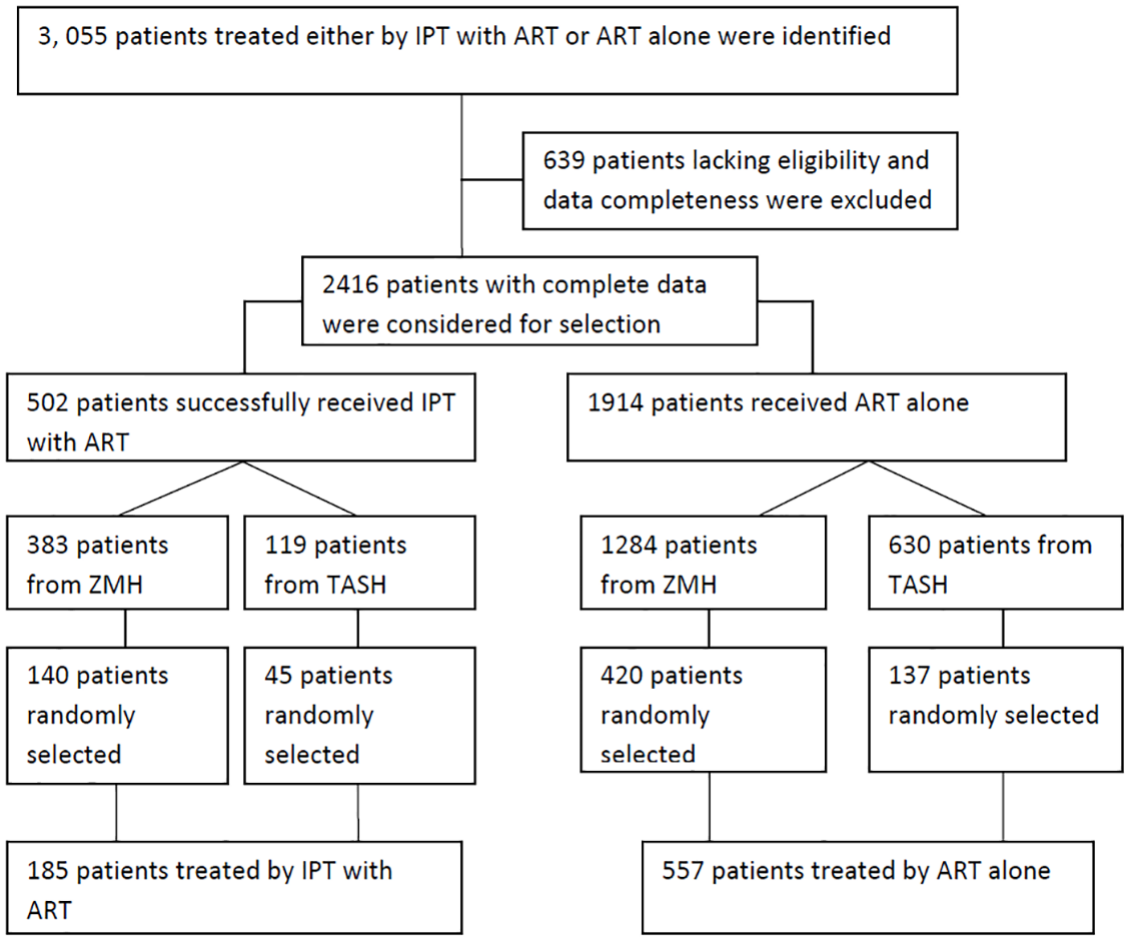
Flow diagram showing selection of study patients on IPT plus ART and ART alone.

The source populations were all PLHIV enrolled into the chronic HIV care follow-up at TASH and ZMH ART clinics. The study participants were selected from a sample of PLHIV enrolled into the chronic HIV care follow-up from September 11, 2009 to March 31, 2012 at the ART clinics. The study participants eligible in the two groups were selected by simple random (lottery) method from the patients enrolled into the two hospitals who received ART or IPT and ART during the specified period. The selection process and the entire work flow of this study are illustrated in Fig 1.

The study was designed and powered to evaluate the key study outcome (all-cause mortality) in 742 PLHIV stratified into two groups; 185 patients as exposed group [those receiving IPT plus ART] and 557 patients as control group [those receiving ART alone]. In this study, it was considered to take α = 0.05 (two-tailed), *β* = 0.20 (power = 80%), control to exposed ratio = 3:1, prevalence of all-cause mortality among controls = 50% (since there was no data of the all-cause mortality prevalence among PLHIV on ART alone in Ethiopia) and minimum risk ratio to detect = 0.75. The sample size of subjects needed in the exposed group (patients on IPT plus ART) was determined to be 165.

Tripling this number, 495 subjects were included in the control group (patients on ART alone). To adjust for missing values, about 10% of the exposed and control subjects were added to yield approximately 185 exposed and 557 control patients. Therefore, the total (overall) study sample size was calculated to be 742 subjects (see, sample size calculation described in Annex 1).

### Data collection process

Identification of the HIV infected patients on IPT and ART versus ART alone was done starting from February, 2013. To obtain complete ascertainment and baseline data of the patients identified at the two HIV care study clinics medical record review was employed. Data was abstracted from individual patient records at the clinics using customized data collection forms. The data abstraction form was extensively piloted before implementation and data collectors were trained to collect the data. Information collected in the data abstraction form include: age, sex, educational levels, dates of HIV diagnosis and treatment initiation, treatment category (ART and IPT or ART alone), baseline histories of opportunistic illnesses (OIs) including tuberculosis, baseline WHO clinical stages, adherence characteristics to ART, addiction or use of drugs (alcohol, tobacco, khat, or shisha), follow-up survival status and time to all-cause mortality if occurred, types of ART regimens, and results of diagnostic tests including CD4 cell counts, liver function tests (LFTs) and creatinine level.

For both all-cause mortality and adherence to ART, and/or IPT verification, follow-up phone calls were made. Patients who were absent from treatment follow-up were asked by repeatedly calling to ensure compliance. During the repeated phone follow-ups, deceased individuals were identified and noted as such on their medical chart. For exact measurement of adherence to ART, both self-report and pharmacy refill records were used. A patient’s follow-up time would end either at his/her last visit to the HIV clinic or at the date of administrative censoring, which was March 31, 2013. Patients who died or were lost to follow-up during the follow-up period were censored on the date of their last clinic visit, as we might not verify the exact dates of death. CD4 cell count data were those reported closest in time to the reference variable.

The quality of data was assured by proper designing and pre-testing of the data abstraction format at ZMH ART clinic on 5% of participants and by giving training to the data collectors before the actual data collection. Every day after data collection, the formats were reviewed and checked for completeness by the investigators.

### Study variables

Age, sex, educational levels and other demographic characteristics, baseline CD4 cell counts, previous TB and other opportunistic illnesses (OIs), baseline WHO clinical stages, use of drugs, adherence characteristics to ART, ART regimen types, and treatment categories (ART only and IPT with ART) were the independent variables. All-cause mortality was the key primary outcome variable measured by the analysis. Time to the all-cause mortality and/or survival rates of patients after initiation with IPT plus ART, or ART alone, was also an outcome variable compared in the study.

### Statistical Analysis

Data were entered into an Epi-info version 3.5.1 and analyzed by the use of SPSS version 20.0. Percent, frequency, median and inter-quartile range (IQR) were the major descriptive statistics employed to summarize baseline demographics and clinical characteristics of the patients. Chi-squared test was also employed to compare proportions in the socio-demographics and clinical characteristics of the patients in the two groups. Kaplan Meier survival analysis was used to compare overall survival rates between the two groups.

Moreover, mortality rates (MR) per 100 person-years (PYs) of follow-up were employed to crudely determine rates of the all-cause mortality. The bivariate and multivariate Cox regression analysis was done to find out the effects of treatment categories, CD4 values, educational levels, use of drugs, baseline WHO clinical stages, and adherence status to ART on the outcome variable (all-cause mortality). In all of the analyses, significance testing was done using two-sided p-values (P) and 95% confidence intervals (95% CI). In all cases, p value <0.05 was considered significant.

### Ethical Consideration

The study was approved by the Addis Ababa University Ethics Review Board of the School of Pharmacy (ERB/SOP/24/05/2013); and Research and Ethics Committee of the Department of Internal Medicine (No. IM/402/2013) at TASH. At ZMH, however, the study approval was done by the Ethical Clearance Committee of Addis Ababa Regional Health Bureau (No. 4240/227). Permission to utilize patient data was obtained officially from the administrative offices of the two hospitals from where the study participants were selected.

## Results

Data were retrospectively collected from the medical records of 742 PLHIV. Data analysis was made in comparison of the two groups of study cohorts; cohort with treatment by IPT plus ART (N = 185) and cohort with treatment by ART alone (N = 557). The total person-year (PY) of follow-up for the entire cohort was 1560 PYs (1160 PYs for patients on ART alone and 400 PYs for patients on IPT plus ART). The median duration of follow-up for ART was 25 months (median duration of follow-up for ART alone group was 25; 26 months for the IPT plus ART group). As shown in Table 1, some baseline characteristics of the two study groups were nearly comparable. More than half of the patients in both groups were females (51.9% and 57.1% for IPT plus ART and ART alone, respectively) and the majority of the patients in the both groups were married (50.3% and 46.4% for IPT plus ART group and ART alone group, respectively). Again, a majority of the patients in the both groups had CD4 cell counts less than or equal to 350 cells/mm^3^ (Table 1). However, the two groups were different with regard to baseline age distribution (P = 0.025), baseline CD4 values (P≤0.001), history of opportunistic infections (P = 0.017) and adherence characteristics to the ART (P = 0.001).

**Table 1:** Baseline demographic and clinical characteristics of patients stratified by exposure categories, Addis Ababa.

Characteristics	Exposure category		P-value
	ART alone	INH and ART	
**Sex (742)—N** **o** **(%)**			0.21
Male	239 (42.9)	89 (48.1)	
Female	318 (57.1)	96 (51.9)	
**Marital Status (726)—N** **o** **(%)**			0.065
Never married	133 (24.1)	43 (24.6)	
Married	261 (46.4)	88 (50.3)	
Divorced	64 (11.6)	11 (6.3)	
Separated	15 (2.7)	11 (6.3)	
Widowed	78 (15.2)	22 (12.5)	
**Educational Status (708)—N** **o** **(%)**			0.67
No education	60 (11.1)	16 (9.4)	
Primary education	161 (29.9)	46 (27)	
Secondary education	235 (43.7)	77 (45.3)	
Higher education	82 (15.3)	31 (18.3)	
**Age (years)—N** **o** **(%)**			0.025*
18–29	131(23.5)	29 (15.7)	
30–39	238 (42.7)	78 (42.2)	
40–49	119 (21.4)	57 (30.8)	
≥50	69 (12.4)	21 (11.3)	
**Baseline CD4 (742)—N** **o** **(%)**			<0.001*
<350 cells/mm3	520 (93.3)	101 (54.6)	
≥350 cells/mm3	37 (6.7)	84 (45.4)	
**Baseline WHO stage (742)—N** **o** **(%)**			0.61
Stage I/II	308 (55.3)	95 (51.4)	
Stage III	195 (35)	69 (37.3)	
Stage IV	54 (9.7)	21 (11.3)	
**Adherence to ART (742)—N** **o** **(%)**			0.001*
Adherent	508 (91.2)	182 (98.3)	
Not adherent	49 (8.8)	3 (1.7)	
**Baseline ART regimen—N** **o** **(%)**			0.058
Preferred 1^st^-line	135 (73)	462 (82.9)	
Alternative 1^st^-line	46 (24.9)	93 (16.7)	
Second line	4 (2.2)	2 (0.40)	
**Previous TB history (679)—N** **o** **(%)**			0.18
Yes	45 (8.9)	10 (5.7)	
No	460 (91.1)	164 (94.3)	
**Previous history of OIs (742)—N** **o** **(%)**			0.017*
Yes	153 (27.5)	68 (36.7)	
No	404 (72.5)	117 (63.3)	
**Alcohol consumption (703)—N** **o** **(%)**			1.26
Yes	125 (23.1)	32 (19.7)	
No	416 (76.9)	130 (80.3)	
**Tobacco smoke (703)—N** **o** **(%)**			0.24
Yes	98 (18.1)	23 (14.2)	
No	443 (81.9)	139 (85.8)	
**Khat chewing (703)—N** **o** **(%)**			0.17
Yes	40 (7.4)	7 (4.3)	
No	501 (92.6)	155 (96.7)	
**Shisha use (703)—N** **o** **(%)**			0.019*
Yes	36 (6.6)	3 (1.8)	
No	505 (93.4)	159 (98.2)	

No, number (count); ART, antiretroviral therapy; IPT, isoniazid preventive therapy; OIs, opportunistic illnesses; TB, tuberculosis

Asterisk (*), shows significant difference

### Mortality Rates

There were 18 versus 116 mortality cases in the patients treated by IPT plus ART, versus ART alone, respectively (Table 2). The overall mortality rate in the cohort was 8.60 per 100 PYs (95% CI 7.12–10.17). Patients who received ART alone had a mortality rate of 10 per 100 PYs (95% CI 8.26–12) while those patients who received IPT plus ART had 4.50 per 100 PYs (95% CI 2.67–7.11).

**Table 2:** Cox regression analysis for associations of all-cause mortality adjusted for covariates, Addis Ababa (N = 669).

Variables	cHR [95% CI]	P value^a^	aHR [95% CI]	P value^a^
**Treatment category**				
ART only	1		1	
IPT plus ART	0.45 [0.37–0.61]	<0.001	0.48 [0.38–0.69]	0.001*
**Baseline CD4**				
<350 cells/mm3	1		1	
≥350 cells/mm3	0.50 [0.28–0.87]	0.015	0.52 [0.28–0.94]	0.033*
**Baseline WHO clinical stage**				
Stage I /II	1		1	
Stage III	1.46 [0.99–2.15]	0.053	1.29 [0.85–1.94]	0.22
Stage IV	2.25 [1.34–3.76]	0.002	2.42 [1.42–4.11]	0.001*
**Educational status**				
No education	1		1	
Primary education	2.07 [1.01–4.26]	0.046	2.20 [1.07–4.52]	0.03*
Secondary education	1.26 [0.62–2.59]	0.52	1.35 [0.66–2.78]	0.40
Higher education	1.59 [0.73–3.47]	0.17	1.76 [0.80–3.84]	0.15
**Adherence status to ART**				
Non-adherent	1		1	
Adherent	0.10 [0.07–0.15]	<0.001	0.12 [0.08–0.20]	<0.001*
**Alcohol consumption**				
No	1		1	
Yes	2.08 [1.43–3.03]	<0.001	1.71 [1.04–2.81]	0.033*
**Tobacco smoke**				
No	1		1	
Yes	2.93 [2.01–4.27]	<0.001	0.87 [0.50–1.52]	0.64
**Khat chewing**				
No	1		1	
Yes	3.28 [2.00–5.36]	<0.001	1.78 [0.94–3.40]	0.07
**Shisha use**				
No	1		1	
Yes	4.44 [2.71–7.28]	<0.001	0.93 [0.40–2.17]	0.87

Asterisk (*) shows significant association; cHR, crude hazard ratio; aHR, adjusted hazard ratio; MR, crude mortality rate

^a^ p values were calculated using the Cox-proportional hazard model

**Fig 2:**
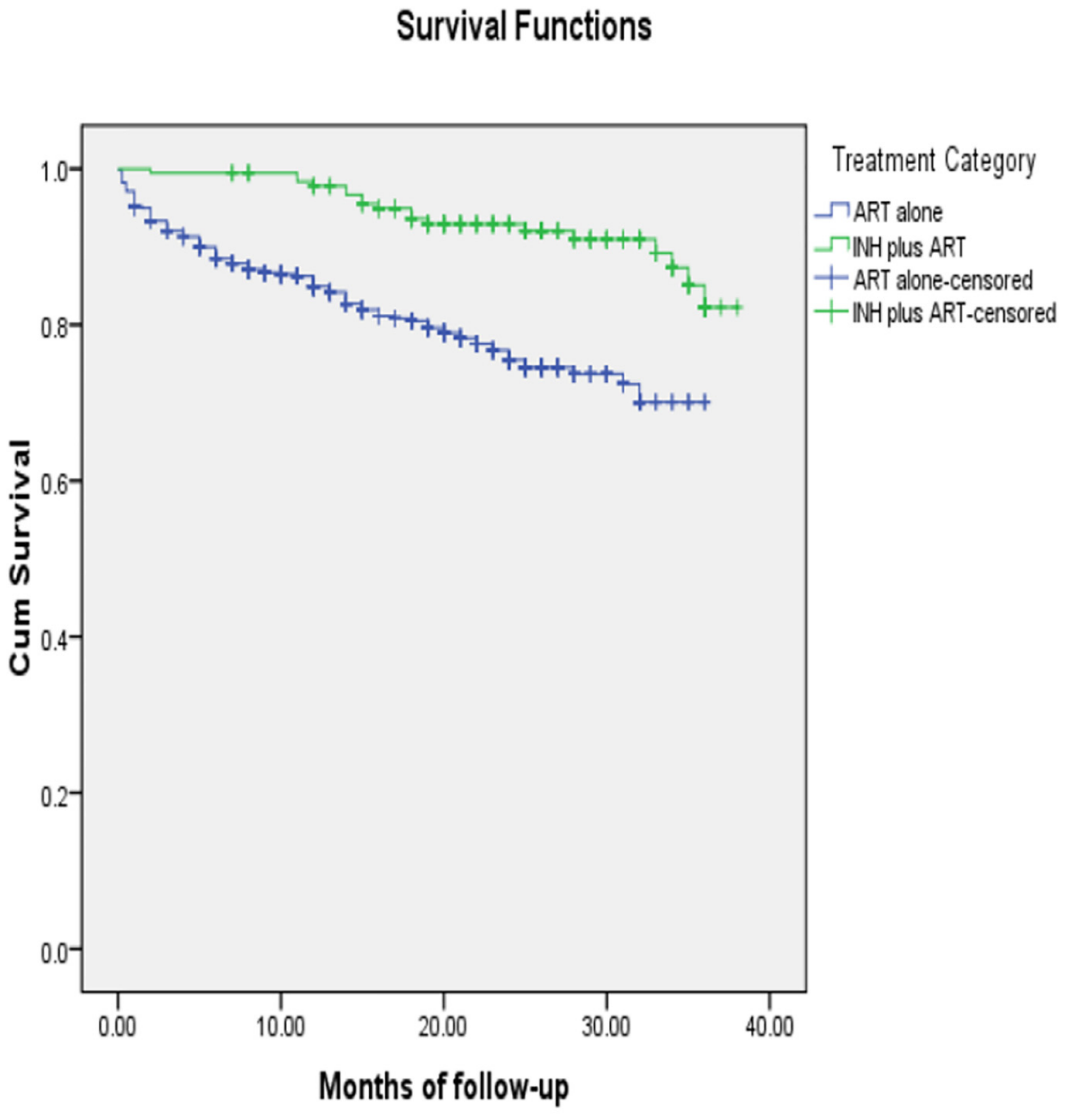
Kaplan Meier survival curve among HIV patients treated by ART alone versus INH plus ART.

A comparison of survival curves of the two treatment groups by Kaplan Meier survival analysis (Fig 2) revealed that the treatment by IPT plus ART provided a longer all-cause mortality protection than the treatment by ART alone (P <0.001). A faster drop in cumulative survival curve of the patients treated by ART alone compared to the survival curve of the patients treated by IPT plus ART showed also that the patients treated by IPT plus ART had higher probabilities of survival than those patients treated by the ART alone (Fig 2).

In Cox regression analysis (Table 2), determination of effect of IPT plus ART on all-cause mortality was made by adjusting for key covariates like CD4 values, WHO clinical stage, educational levels, adherence status to ART and use of drugs. Accordingly, both unadjusted and adjusted Cox regression analyses showed significantly reduced hazards of all-cause mortality in patients treated with IPT plus ART (crude hazard ratio [cHR] 0.45; 95% CI 0.37–0.61 and adjusted hazard ratio [aHR] 0.48; 95% CI 0.38–0.69). Several other factors were also found to affect the mortality besides the treatment group (Table 2). As shown in Table 2, WHO stage IV (aHR 2.42: 95% CI 1.42–4.11), CD4 values ≥350cells/mm^3^ (aHR 0.52; 95% CI 0.28–0.94), adherence to ART (aHR 0.12; 95% CI 0.08–0.20), primary levels of education (aHR 2.20; 95% CI 1.07–4.52); and alcohol consumption (aHR 1.71; 95% CI 1.04–2.81) were the factors strongly associated with the all-cause mortality.

## Discussion

This comparative study found a significant impact of the use of IPT (for 6 months) plus ART on the reduction of all-cause mortality as compared to ART alone. The effect of the treatment by IPT plus ART on the delay of time to the all-cause mortality was also promising. In the analysis, the likelihood of the all-cause mortality was higher in PLHIV who had CD4 values <350 cells/mm^3^; were ART non-adherent; and were exposed to WHO clinical stage IV, with primary levels of education, and were alcohol users. In addition to the treatment category, therefore, CD4 values, WHO clinical stages, adherence status to ART, educational levels, and alcohol use were factors associated with the all-cause mortality and discussed separately.

The treatments of HIV patients by IPT and ART compared to ART alone reduced unadjusted mortality rates from 10 cases per 100 PYs to 4.5 cases per 100 PYs of follow-up in the HIV-infected patients at ZMH and TASH. After adjustment for CD4 values, WHO clinical stage, adherence to ART, educational status, and use of drugs were made, the treatment by IPT and ART reduced the likelihood of all-cause mortality by 52% compared to the treatment by ART alone. Similarly, in a cohort study in South Africa, the unadjusted mortality rate was lower among PLHIV who received IPT as compared to those who did not receive IPT (3.7 /100 PYs vs. 11.1/100 PYs, respectively); this was associated with an adjusted 49% overall mortality rate reduction by the IPT (aHR 0.51, 95% CI 0.32–0.80) [15]. Two meta-data and a randomized controlled trial also revealed a TB-related mortality reduction by TB preventive therapies [10, 16, 17]. However, in the same meta-data, all-cause mortality reduction was not significant [10]. Moreover, though the all-cause mortality reduction was not evidenced, the mortality benefits of TB preventive therapies, including the IPT, was shown by a study in Uganda [18].

Furthermore, another study in South Africa also showed that isoniazid prophylaxis in HIV- infected children on ART was associated with a 54% reduction in the all-cause mortality [19]. This mortality reduction may not exactly explain the mortality rate in adults, but it is supported by another study that explained a better mortality reduction when antiretroviral drugs are utilized in combination with the tuberculosis preventive therapy [20]. Additionally, a placebo controlled randomized trial of IPT prophylaxis in adult PLHIV with-or-without ART showed a significantly reduced hazard of death [21]. Literature also shows the potential of IPT to reduce illness and death especially among PLHIV regardless of ART [22]. On the other hand, an increased hazard of tuberculosis on mortality in PLHIV was described by a similar study [23] as HIV-related tuberculosis is the leading infectious cause of death world-wide [24]. However, this may not justify the all-cause mortality. Most of the randomized controlled trials of IPT included in recent meta-analysis did not involve ART and the effect of IPT on the all-cause mortality was not evidenced by these studies [18, 25, 26]. However, the addition of IPT to ART may enhance adherence to ART. This could be due to the fact that IPT follow-up is made in accordance with the implementation of directly observed treatment short course (DOTS) and such strategies are lacking when the patient is on ART alone. The greater adherence rate in the IPT plus ART group compared to the ART alone group in our study could, therefore, escalate the survival benefit of IPT plus ART. Besides, the retrospective nature of our data could also contribute to the partial inconsistence between the results of our study and findings of some prospective studies.

Accordingly, despite the fact that IPT significantly reduced the deaths caused by tuberculosis disease, the all-cause mortality reduction by IPT was not statistically significant in some literatures [27, 28]. The evidence of all-cause mortality reduction by the preventive therapy was also contradicted by another study, although a favorable trend is found in HIV patients positive for tuberculin test [29]. However, the higher baseline CD4 values in the patients treated by IPT and ART compared to the ART alone in our cohort could contribute to the lower death rates in the IPT plus ART treated patients. In our study, HIV patients with CD values ≥350 cells/mm^3^ had 0.52 times lower rates of all-cause mortality adjusted for covariates compared to the patients who had CD4 values <350 cells/mm^3^. Previous studies also showed that having lower CD4 values at initiation of ART is found to be a strong predictor of death [30–34]. In addition to the treatment category and CD4 values, baseline WHO stages, adherence characteristics to ART, educational levels, and use of substances were covariates and as such included in the final multivariable analyses.

The association between WHO clinical stage IV and occurrence of the all-cause mortality was significant. Compared to PLHIV who were on WHO clinical stage I/II, PLHIV who were on WHO clinical stage IV at baseline had 2.42 times higher hazards of the all-cause mortality. Consistent to this finding, some literatures also showed increased hazards of death in patients with AIDS symptoms at baseline [32, 34, 35].

Compared to PLHIV with characteristics of ART non-adherence, PLHIV who were treatment adherent had 0.12 times lower hazards of the all-cause mortality. This association of ART non-adherence with the increased likelihood of mortality was also shown by a study that compared compliance and mortality in Malawi [36]. Moreover, the high adherence to ART recorded in survivors of cohort studies also implied the association of non-adherence with the occurrence of death [33, 37, 38].

The association between educational levels and the likelihood of death in reference to patients with no formal education did not reach significant levels except for the patients at primary levels of education. Compared to PLHIV who had no formal education, the PLHIV who were at a primary level of education had 2.20 times higher likelihood of death. This could be explained by the non-adherence of patients at lower levels of formal education as under two years of formal education and less than college education were found to be predictors of non-adherence [39, 40].

Moreover, except for alcohol consumption, the effects of use of tobacco, khat and shisha on the incidence of mortality in HIV patients were not statistically significant. However, compared to PLHIV who did not use alcohol, PLHIV who consumed alcohol had a 1.71 times higher likelihood of death. Similarly, hazardous alcohol use is independently associated with decreased ART utilization in a Baltimore, MD, USA study [41]—and this could lead to an increased likelihood of death. In addition, additional literature also illustrates that alcohol consumption reduces compliance with ART regimens resulting in premature mortality. As such, alcohol and HIV modulate innate and adaptive immunity and alcohol consumption for PLHIV increases the likelihood of viral replication and leads to increased susceptibility to contract opportunistic infections and other co-morbid conditions [42].

Though lengthy follow-ups were made to ensure reliable outcome ascertainment, there were some limitations to this analysis. First, data of this study were abstracted from medical records and may suffer from missing information, or variable deficiencies that could result in under- or over-estimations of all-cause mortality. In light with this, adherence to ART was not measured by direct observation and this may result in inaccurate estimation of the adherence rate. Secondly, measurement of specific disease caused death was not done and only all-cause mortality was considered. The third limitation is related to the measurement of sensitive predictors like drug use, which could be inaccurately estimated without the aid of prospective observational interventions. Confounding effects of baseline CD4 values, baseline WHO clinical stage and adherence status to ART were adjusted for during analysis by Cox regression; however, confounding effects of unmeasured variables (weight, hemoglobin value, etc.) could not be adjusted for in the analysis. Lastly, it should be noted that there could be some underrepresentation of patients whose HIV disease progressed rapidly, and thus died before becoming eligible to treatment as the study was a prevalent cohort. Therefore, interpretation of the findings in this analysis should be made in consideration of these limitations.

## Conclusion

The present comparative study clearly demonstrated the effects of IPT plus ART on the reduction of rates of all-cause mortality compared to treatment by ART alone in PLHIV. In addition, the study found non-adherence to antiretroviral drugs treatment, CD4 values <350 cells/mm3, WHO clinical stage IV, primary level of education, and alcohol consumption as strong predictors of increased likelihood of the all-cause mortality. From the findings, therefore, wider use of IPT plus ART under conditions of good adherence, more education and limited drug and alcohol use could significantly reduce the rates of mortality in PLHIV.

## Appendix

### Annex 1. Sample size calculation

$$N=\frac{1}{{\left[P\left(1-R\right)\right]}^{2}}{\left[Z_{1-\alpha \;}\sqrt{\left(1+\;\frac{1}{K}\right)U\left(1-U\right)}+\;Z_{\left(1-\beta \right)\;}\sqrt{PR\left(1-PR\right)+\;\frac{P\left(1-P\right)}{K}}\right]}^{2}$$

Where, P is the prevalence of mortality in the controls; R is the minimum risk ratio to be detected; α is the type I error rate which is acceptable; β is the type II error rate which is acceptable; Z_1- α_ and Z_1−β_ refer to the unit normal deviates corresponding to α and β; K is the ratio of number of control subjects to the number of exposed subjects; and

$$U=\frac{KP+PR}{K+1}$$

### Annex 2. Abbreviations (Acronyms)

3TC: Lamivudine
ABC: Abacavir
ATV/r: Atazanavir boosted with ritonavir
d4T: Stavudine
ddI: Didanosine
EFV: Efavirenz
LPV/r: Lopinavir boosted with ritonavir
NVP: Nevirapine
TDF: Tenofovir
ZDV: Zidovudine

## Supporting Information

S1 Fig Kaplan Meier survival curve among HIV patients with average follow-up CD4 < 350 cells/mm3 versus CD4 ≥ 350 cells/mm3 at TASH and ZMH, September 11, 2009—March 31, 2013. (PDF)

S1 Protocol Individual patient data abstraction form for HIV-infected patients on IPT plus ART and ART alone versus effects on all-cause mortality, incident TB and HIV progression, Addis Ababa, Ethiopia. (PDF)

S1 Table Characteristics of HIV-infected patients on IPT plus ART or ART alone survived or died during the study period, Addis Ababa, Ethiopia. (DOCX)
