# Supplementary figures and images for: A Description of Mortality Associated with IPT plus ART Compared to ART Alone among HIV-Infected Individuals in Addis Ababa, Ethiopia: A Cohort Study
Source: PLoS One. 2015 Sep 8;10(9):e0137492. doi: 10.1371/journal.pone.0137492 (PMC4562624; doi:10.1371/journal.pone.0137492)

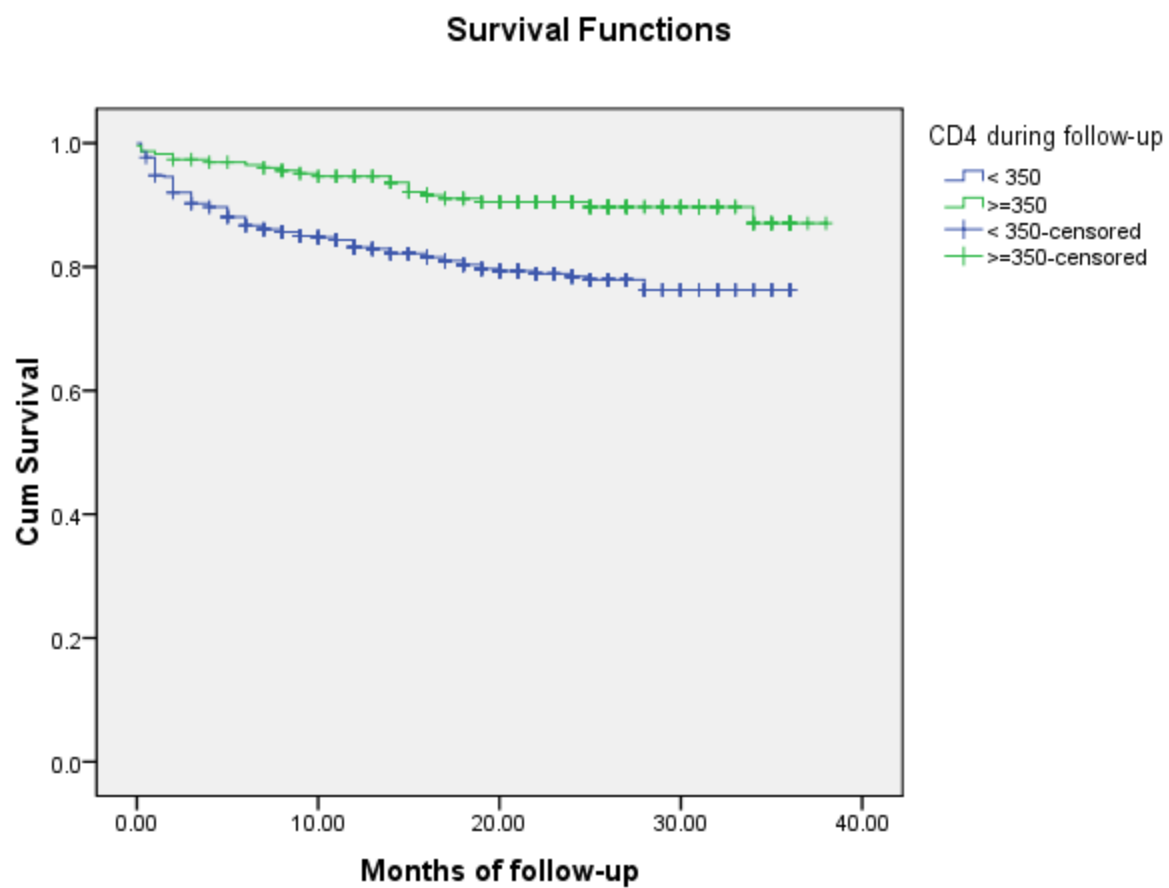

S1 Fig.

Supplement: S1 Fig — (PDF) [file pone.0137492.s001.pdf]
