# Supplementary material for: A Description of Mortality Associated with IPT plus ART Compared to ART Alone among HIV-Infected Individuals in Addis Ababa, Ethiopia: A Cohort Study
Source: PLoS One. 2015 Sep 8;10(9):e0137492. doi: 10.1371/journal.pone.0137492 (PMC4562624; doi:10.1371/journal.pone.0137492)
Supplement: S1 Protocol — (PDF) [file pone.0137492.s002.pdf]

S1 Protocol

1. Sociodemographic characteristics of patient

1.1. ART unique number (ID)_________________; Medical registration number_________

1.2. AIDS diagnosis/confirmation date__________________

1.3. Sex: a. male b. female

1.4. Marital status: a. never married b. married c. divorced d. widowed e. separated

1.5. Educational status: a. No education b. primary c. secondary d. tertiary

1.6. Drug Addiction: a. Alcohol b. Tobacco c. Khat d. Shisha e. None f. Others, specify______________________

1.7. Care giver/HIV status exposed to: a. husband/wife b. Father c. Mother d. brother/sister e. own child f. Relatives

2. Clinical characteristics of patient

2.1. ART start date_________________

2.2. IPT start date __________________

2.3. Baseline weight (Kg)___________

2.4. Age at baseline (Yr): a. < 30 (_____) b. 30 – 39 (_____) c. 40 – 49 (_____) d. ≥50 (_____)

2.5. Previous Tuberculosis: a. yes, specify date treatment completed, __/__/___ b. No

2.6. Documented Other OIs: a. Yes, specify____________________________ b. No

2.7. Baseline CD4 cells count (cells/mm3): a. < 200 (_______) b.200–349 (_______) c. 350 – 499 (_________) d. ≥ 500 (_________)

2.8. Hemoglobin level at baseline (mg/dl) ___________

2.9. ALT (SGPT) level at baseline ___________

2.10. AST (SGOT) level at baseline ___________

2.11. BUN level at baseline ___________

2.12. Creatinine level at baseline ___________

2.13. WHO clinical stage of AIDS at baseline: a. I b. II c. III d. IV

2.14. Baseline ART regimen: a. 1a (preferred 1st line) b. 1b (alternative 1st line) c. 2 (second-line)

2.15. Adherence status during follow-up period: a. good b. fair c. poor
d. lost to follow-up

2.16. Living status of the patient during follow-up: a. alive b. died, specify date ____/____/_____

2.17. If died, what was the cause?

a. TB
b. OIs, specify_________
c. Unknown

2.18. Tuberculosis diagnosed during follow-up period: a. No b. Yes, specify date ____/_____/_____

2.19. New AIDS stage III/IV or persisting stage IV: a. No b. Yes, specify date ____/_____/_____

2.20. Any document of adverse event/side effect: a. No b. Yes, specify__________ date ____/____/_____

3. Outcome variables of treatment (IPT plus ART or ART alone) at each follow up (3, 6, 9, 12, 18, 24, and 30 months)

**Variable**	**3rd month**	**6th month**	**9th month**	**12th Mo**	**18th Mo**	**24th Mo**	**30th Mo**
Death							
Active TB diagnosed							
New AIDS (III/IV)							
New adverse event							
CD4 (cells/mm3)							
Hgb (mg/dl)							
AST (SGOT)							
ALT (SGPT)							
BUN							
Creatinine							

Note: **1a = Preferred First- line regimens**

- TDF+3TC/FTC+EFV = triple Fixed Drug Combination (FDC)
- ZDV+3TC+EFV= double FDC +EFV
- ZDV+3TC+NVP = triple FDC

1b = Alternative First-line regimens

- D4T/3TC/EFV = double FDC (d4T/3TC) + EFV
- TDF/3TC/NVP
- D4T/3TC/NVP = triple FDC Or ABC/3TC/EFV
- ABC/3TC/NVP
- ABC/3TC/ZDV = double FDC + ABC

2= second line regimens

- ZDV ± 3TC +LPV/r or ATV/r
- ZDV+ABC+LPV/r or ATV/r
- TDF+3TC ± ZDV+LPV/r or ATV/r
- ABC + ddI +LPV/r or ATV/r
- EFV or NVP + LPV/r or ATV/r
