# Supplementary material for: A Description of Mortality Associated with IPT plus ART Compared to ART Alone among HIV-Infected Individuals in Addis Ababa, Ethiopia: A Cohort Study
Source: PLoS One. 2015 Sep 8;10(9):e0137492. doi: 10.1371/journal.pone.0137492 (PMC4562624; doi:10.1371/journal.pone.0137492)
Supplement: S1 Table — (DOCX) [file pone.0137492.s003.docx]

## S1 Table

| **Characteristics** | **Alive** | **Died** | **P value** |
| --- | --- | --- | --- |
| Sex⎯No (%) |  |  | 0.36 |
| Male | 264 (80.5) | 64 (19.5) |  |
| Female | 344 (83.1) | 70 (16.9) |  |
| Marital status (726) ⎯No (%) |  |  | 0.19 |
| Never married | 134 (76.1) | 42 (23.9) |  |
| Married | 293 (84.0) | 56 (16.0) |  |
| Divorced | 62 (82.7) | 13 (17.3) |  |
| Separated | 23 (88.5) | 3 (11.5) |  |
| Widowed | 84 (84.0) | 16 (16.0) |  |
| Educational status (708) ⎯No (%) |  |  | 0.27 |
| No education | 65 (85.5) | 11 (14.5) |  |
| Primary education | 162 (78.3) | 45 (21.7) |  |
| Secondary education | 263 (84.3) | 49 (15.7) |  |
| Higher education | 91 (80.5) | 22 (19.5) |  |
| Baseline Age (Years) ⎯No (%) |  |  | 0.60 |
| 18-29 | 129 (80.6) | 31 (19.4) |  |
| 30-39 | 262 (82.9) | 54(17.1) |  |
| 40-49 | 140 (79.5) | 36 (20.5) |  |
| ≥50 | 77 (85.6) | 13 (14.4) |  |
| Previous history of tuberculosis(679) |  |  | 0.75 |
| No | 510 (81.7) | 114 (18.3) |  |
| Yes | 44 (80.0) | 11 (20.0) |  |
| Previous history of OIs (742) |  |  | 0.98 |
| No | 427 (82.0) | 94 (18.0) |  |
| Yes | 181 (81.9) | 40 (18.1) |  |
| Baseline CD4 (742)⎯No (%) |  |  | 0.13 |
| <350 cells/mm3 | 503 (82.6) | 118 (17.4) |  |
| ≥350 cells/mm3 | 105 (86.8) | 16 (13.2) |  |
| Baseline ART regimen type |  |  | 0.45 |
| Preferred 1^st^-line | 484 (81.1) | 113 (18.9) |  |
| Alternative 1^st^-line | 119 (85.6) | 20 (14.4) |  |
| Second line | 5 (83.3) | 1 (16.7) |  |
| WHO Clinical stages of AIDS |  |  | <0.001* |
| Stage I/II | 347 (86.1) | 56 (13.9) |  |
| Stage III | 210 (79.5) | 54 (20.5) |  |
| Stage IV | 51 (68.0) | 24 (32.0) |  |
| Adherence Status |  |  | <0.001* |
| Adherent | 594 (86.1) | 96 (13.9) |  |
| Non-adherent | 14 (26.9) | 38 (73.1) |  |
| Alcohol addiction (703)⎯No (%) |  |  | <0.001* |
| Yes | 113 (72.0) | 44 (28.0) |  |
| No | 462 (84.6) | 84 (15.4) |  |
| Tobacco addiction (703)⎯No (%) |  |  | <0.001* |
| Yes | 76 (62.8) | 45 (37.2) |  |
| No | 499 (85.7) | 83 (14.3) |  |
| Khat addiction (703)⎯No (%) |  |  | <0.001* |
| Yes | 27 (57.4) | 20 (42.6) |  |
| No | 548 (83.5) | 108 (16.5) |  |
| Shisha addiction (703)⎯No (%) |  |  | <0.001* |
| Yes | 19 (48.7) | 20 (51.3) |  |
| No | 556 (83.7) | 108 (16.3) |  |

## *Asterisk (*), shows significant difference*
